# Metabolomic profiling of plasma from middle-aged and advanced-age male mice reveals the metabolic abnormalities of carnitine biosynthesis in metallothionein gene knockout mice

**DOI:** 10.18632/aging.203731

**Published:** 2021-12-01

**Authors:** Yoshito Kadota, Asuka Yano, Takashige Kawakami, Masao Sato, Shinya Suzuki

**Affiliations:** 1 Faculty of Pharmaceutical Sciences, Tokushima Bunri University, Tokushima 770-8514, Japan

**Keywords:** metallothionein, metabolomic profiling, L-carnitine, N6,N6,N6-trimethyl-L-lysine, tmlhe gene

## Abstract

Metallothionein (MT) is a family of low molecular weight, cysteine-rich proteins that regulate zinc homeostasis and have potential protective effects against oxidative stress and toxic metals. *MT1* and *MT2* gene knockout (MTKO) mice show shorter lifespans than wild-type (WT) mice. In this study, we aimed to investigate how MT gene deficiency accelerates aging. We performed comparative metabolomic analyses of plasma between MTKO and WT male mice at middle age (50-week-old) and advanced age (100-week-old) using liquid chromatography with time-of-flight mass spectrometry (LC-TOF-MS). The concentration of *N*6,*N*6,*N*6-trimethyl-L-lysine (TML), which is a metabolic intermediate in carnitine biosynthesis, was consistently higher in the plasma of MTKO mice compared to that of WT mice at middle and advanced age. Quantitative reverse transcription PCR (RT-PCR) analysis revealed remarkably lower mRNA levels of *Tmlhe*, which encodes TML dioxygenase, in the liver and kidney of male MTKO mice compared to that of WT mice. L-carnitine is essential for β-oxidation of long-chain fatty acids in mitochondria, the activity of which is closely related to aging. Our results suggest that reduced carnitine biosynthesis capacity in MTKO mice compared to WT mice led to metabolic disorders of fatty acids in mitochondria in MTKO mice, which may have caused shortened lifespans.

## INTRODUCTION

Population aging is predicted to become a major global public health challenge in the next decade, as the advanced age group population has been increasing [1]. The principal theories of aging are fraught with complications [2]. López-Otín C et al. have characterized aging as a progressive loss of physiological integrity, leading to impaired function and increased vulnerability to death [3]. They proposed nine candidate hallmarks that contribute to the aging process and together determine the aging phenotype; 1) genomic instability, 2) telomere attrition, 3) epigenetic alterations, 4) loss of proteostasis, 5) deregulated nutrient-sensing, 6) mitochondrial dysfunction, 7) cellular senescence, 8) stem cell exhaustion, and 9) altered intercellular communication. In addition, these hallmarks were grouped into three categories, which may suggest the order in which the hallmarks occur. Nevertheless, it is difficult to quantify and evaluate aging because the principal theories of aging are intricately intertwined. To overcome these difficulties, we must discover age-dependent changes occurring during various ages, which may provide new information and opportunities to understand the mechanisms of aging. Metabolomics, using blood, serum, and plasma, has emerged as a powerful tool to characterize organism phenotypes, and identify altered metabolites, pathways, and novel biomarkers in aging and disease, which will offer wide clinical applications [4, 5].

Metallothionein (MT) family proteins are low-molecular-weight cysteine-rich proteins induced by various chemical and physical stresses that produce reactive oxygen species (ROS) [6]. MTs play a role in zinc homeostasis and have potential protective effects against free radicals (e.g., hydroxyl radicals) and toxic metals (e.g., cadmium and mercury ions) [7]. Increased cellular abundance of MTs is associated with biological outcomes of cell viability, immune function, mitochondrial respiration, neuroprotection, and fat mass regulation, which are directly linked to aging [8]. We have shown that MTs protect adipose tissue from environmental stress and that MTs play a role in maintaining the appropriate size of adipocytes [9, 10]. The possible role of MTs in pro-longevity interventions has been elucidated in many species. Elevated *MT* gene expression has been observed in tissues from long-lived mice and worms [8, 11]. In rodents, *MT*-transgenic mice have extended lifespans [12, 13]. In humans, *MT* polymorphisms are associated with longevity [14–17]. However, *MT1* and *MT2* knockout (MTKO) mice were exclusively used to study the sensitivity and lethality of toxic substances, including cadmium [18] and cisplatin [19], compared with wild-type (WT) mice. The MTKO mice were used to elucidate the role of MT, because *MT* genes are induced in response to multiple stresses and exert protection against these stresses. However, it was unknown whether *MT* gene deficiency affects lifespan in an ordinary lifestyle without anthropogenic stressors. Our previous study showed that MT deficiency shortened the lifespan of 129sv strain mice of both sexes, and the median lifespans of male MTKO mice and WT mice were 106 and 129 weeks, respectively [20]. Older male MTKO mice that lived beyond the mean lifespan exhibited signs of excessive and extraordinary symptoms of senescence along with a drastic reduction in body weight. The reduction in the body weight of male MTKO mice started at approximately 50 weeks, which coincides with the first incidence of mortality in male MTKO mice; the first death of male MTKO and WT mice was observed at 55 weeks and 70 weeks, respectively [20]. Metabolic changes that affect longevity and aging may have occurred around this time in MTKO mice.

In this study, we performed a metabolomic analysis of plasma in middle-aged (50-week-old: the age at which the body weight of male MTKO mice began decreasing, and immediately before the first death in MTKO mice) and advanced age (100-week-old; close to the median lifespan of male MTKO mice) mice using liquid chromatography time-of-flight mass spectrometry (LC-TOF-MS), to obtain information regarding altered phenotypic profiles of both MTKO and WT male mice, and to determine the biomarkers of aging accelerated by the deficiency of *MT* genes.

## RESULTS

### Multivariate statistical analyses

**Figure 1:**
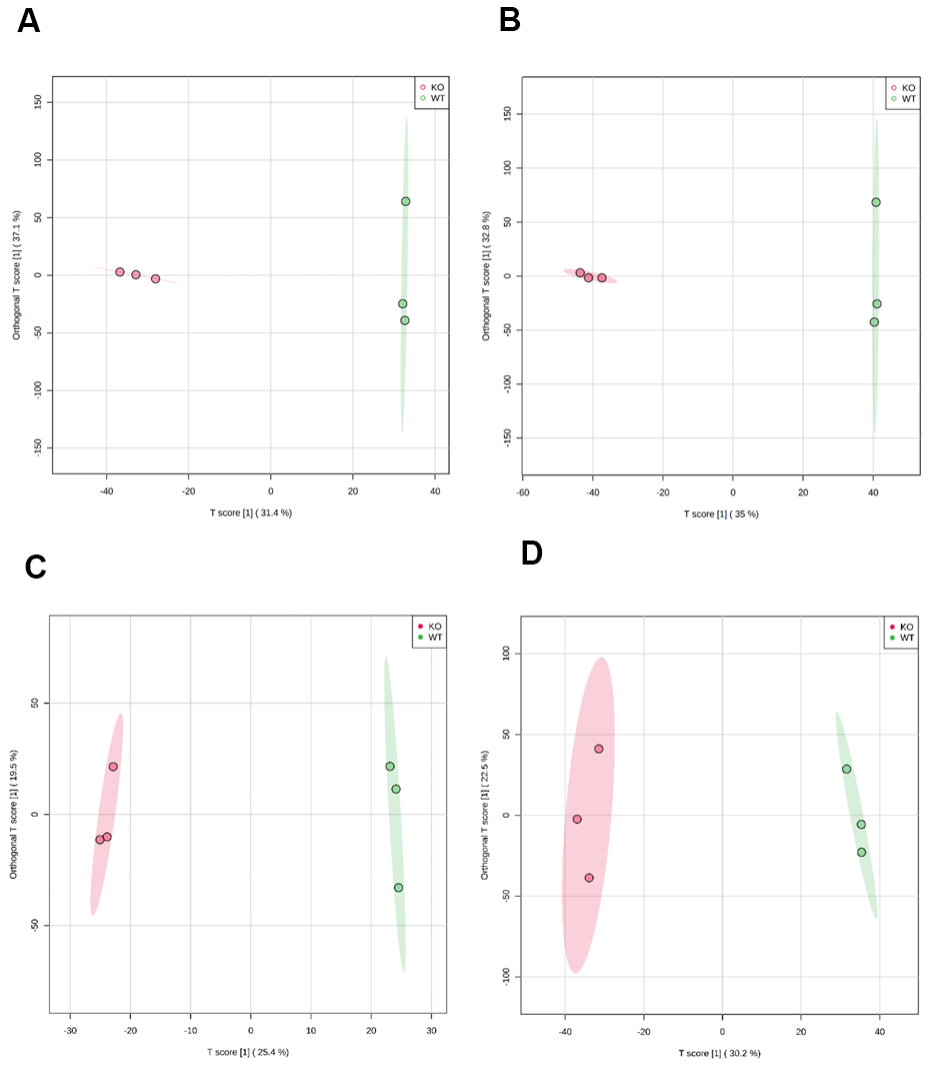
**Scatter plot of scores based on the OPLS-DA model.** (**A**) ESI^+^ scan in 50-week-old mice; (**B**) ESI^-^ scan in 50-week-old mice; (**C**) ESI^+^ scan in 100-week-old mice; (**D**) ESI^-^ scan in 100-week-old mice. Magenta circles indicate MTKO mice and green circles indicate WT mice.

Representative base peak intensity (BPI) chromatograms were obtained from plasma in electrospray ionization positive (ESI^+^) and negative (ESI^-^) modes, respectively (Supplementary Figure 1A, 1B). All peaks in ESI^+^ or ESI^-^ were merged and imported into the SIMCA-P software for multivariate statistical analysis. To investigate global metabolism variations, we first used principal component analysis (PCA) to analyze all observations acquired in both ion modes. As shown in the PCA plot (Supplementary Figure 2), an overview of all samples in the data can be observed and exhibits an unclear grouping trend between the two groups. To eliminate any non-specific effects of the operative technique and confirm the biomarkers, partial least squares discriminant analysis (PLS-DA) and orthogonal partial least squares-discriminant analysis (OPLS-DA) were used to compare metabolic changes between the two groups. In the PLS-DA score plot (Supplementary Figure 3) or OPLS-DA score plot (Figure 1A–1D), a clear separation of the two groups was observed. Univariate analyses, including fold change (FC) analysis and *t*-test, were performed on a volcano plot (Supplementary Figure 4). We selected metabolites with variable importance in projection (VIP) > 1.5, FC > 2.0, and *P* < 0.05 as significant compounds.

### Quantitative comparison of metabolites in the plasma between 50-week-old MTKO and WT male mice

### Identification of metabolites in the plasma

We identified 599 differential metabolites, of which 242 were detected in the positive ion mode and 357 in the negative ion mode in the plasma of 50-week-old mice. The results of the hierarchical clustering analysis (HCA) of compounds with statistically significant differences between the groups of MTKO and WT male mice are summarized in Table 1. The levels of 19 metabolites were higher, whereas those of 27 others were lower, in the plasma of MTKO mice compared to that of WT mice.

**Table 1:** HCA data; identified metabolites showing a difference between MTKO and WT male group under ESI^-^ and ESI^+^ scans of 50-week-old mice (*n* = 3).

**No.**	**Compound name**	**Molecular formula**	**RT^a^ [min]**	**Monoisotopic mass**	**Delta (ppm)**	**FC^b^**	***P*-value**	**VIP^c^**
**Positive ion (ESI+)**								
1	L-Lactic acid	C3H6O3	1.20	90.0317	5	0.317	0.006	1.68
2	Oxoglutaric acid	C5H6O5	1.22	146.0215	3	0.283	0.005	1.68
3	Citric acid	C6H8O7	1.23	192.0270	2	0.394	0.002	1.72
4	Glycerol 3-phosphate	C3H9O6P	1.12	172.0137	3	0.363	0.000	1.77
5	Taurine	C2H7NO3S	1.15	125.0147	3	0.349	0.038	1.50
6	L-Phenylalanine	C9H11NO2	0.82	165.0790	2	2.540	0.026	1.55
7	*N*-Lactoylphenylalanine	C12H15NO4	5.48	237.1001	2	0.270	0.016	1.61
8	*N*6,*N*6,*N*6-Trimethyl-L-lysine	C9H20N2O2	0.85	188.1525	2	3.379	0.035	1.51
9	Uracil	C4H4N2O2	1.22	112.0273	5	0.463	0.006	1.67
10	Deoxycytidine	C9H13N3O4	0.99	227.0906	1	3.036	0.000	1.78
11	Xanthine	C5H4N4O2	1.78	152.0334	2	0.031	0.001	1.75
13	Xanthosine	C10H12N4O6	1.78	284.0757	2	0.033	0.002	1.73
14	Azelaic acid	C9H16O4	6.07	188.1049	2	0.384	0.029	1.53
15	Eicosapentaenoic acid	C20H30O2	13.24	302.2246	2	0.173	0.026	1.55
16	Arachidonic acid	C20H32O2	13.61	304.2402	1	0.207	0.025	1.56
17	Prostaglandin E2	C20H32O5	8.26	352.2250	5	0.102	0.010	1.64
18	CPA(16:0/0:0)	C19H37O6P	15.77	392.2328	2	0.305	0.034	1.51
19	LysoPC(10:0/0:0)	C19H39O7P	8.21	410.2433	2	2.396	0.025	1.55
20	LysoPE(0:0/14:0)	C19H40NO7P	8.89	425.2542	2	2.657	0.013	1.62
21	LysoPE(16:0/0:0)	C21H44NO7P	10.26	453.2855	1	2.383	0.026	1.55
22	LysoPC(16:0/0:0)	C24H50NO7P	11.34	495.3325	1	2.178	0.003	1.72
23	Glycocholic acid	C26H43NO6	12.74	465.3090	2	0.223	0.000	1.77
24	Taurocholic acid	C26H45NO7S	7.06	515.2917	15	0.230	0.014	1.62
25	Glycochenodeoxycholate-3-sulfate	C26H43NO8S	4.56	529.2709	31	0.134	0.005	1.69
12	Bilirubin	C33H36N4O6	8.08	584.2635	0	5.342	0.007	1.66
26	4-Hydroxybenzaldehyde	C7H6O2	0.61	122.0368	26	0.360	0.001	1.73
27	Indole-3-carboxaldehyde	C9H7NO	0.61	145.0528	2	0.271	0.001	1.74
28	DL-2-Aminooctanoic acid	C8H17NO2	1.23	159.1259	2	3.021	0.000	1.77
**Negative ion (ESI-)**								
29	L-Lactic acid	C3H6O3	0.82	90.0317	16	0.445	0.018	1.50
30	L-Lysine	C6H14N2O2	0.76	146.1055	9	2.086	0.019	1.50
31	Histidylglutamic acid	C11H16N4O5	1.31	284.1121	6	2.328	0.013	1.54
32	Xanthosine	C10H12N4O6	1.76	284.0757	1	0.065	0.001	1.65
33	Hexadecanedioic acid	C16H30O4	8.70	286.2144	1	2.391	0.001	1.66
34	12,13-DHOME	C18H34O4	14.29	314.2457	1	2.878	0.002	1.63
35	12-HETE	C20H32O3	13.93	320.2351	1	0.087	0.009	1.56
36	15-Keto-13,14-dihydroprostaglandin A2	C20H30O4	11.12	334.2144	1	0.021	0.014	1.53
37	12(*S*)-HPETE	C20H32O4	10.59	336.2301	1	0.488	0.016	1.51
38	Prostaglandin E2	C20H32O5	8.56	352.2250	8	0.020	0.014	1.53
39	Thromboxane B2	C20H34O6	8.17	370.2355	1	0.179	0.001	1.65
40	LysoPE(0:0/20:3(5*Z*,8*Z*,11*Z*))	C25H46NO7P	11.33	503.3012	1	2.629	0.000	1.69
41	LysoPE(0:0/24:6(6*Z*,9*Z*,12*Z*,15*Z*,18*Z*,21*Z*))	C29H48NO7P	12.17	553.3168	2	2.313	0.002	1.64
42	PG(16:1(9*Z*)/16:0)	C38H73O10P	19.12	720.4941	1	8.253	0.008	1.57
43	PS(22:0/15:0)	C43H84NO10P	9.47	805.5833	4	0.332	0.003	1.62
44	Bilirubin	C33H36N4O6	8.07	584.2635	1	7.311	0.004	1.60
45	Dehydrochorismic acid	C10H8O6	13.40	224.0321	13	2.164	0.012	1.54
46	Hydroxystilbamidine	C16H16N4O	8.25	280.1324	6	3.681	0.002	1.63

RT^a^, retention time; FC^b^, fold change in the comparison (average in MTKO mice/WT mice); VIP^c^, variable importance in the projection scores derived from discriminant analysis. The metabolites were filtered and confirmed by combining the results of the VIP values (VIP > 1.5), FC values (FC > 2.0), and t-test (*P* < 0.05).

Abbreviations: 12,13-DHOME, (12,13-dihydroxy-9*Z*-octadecenoic acid); 12-HETE, (12-hydroxy-eicosatetraenoic-acid); 12(S)-HPETE, (12*S*-hydroperoxy-5*Z*, 8*Z*, 10*E*, 14*Z*-eicosatetraenoic acid); PE, (phosphatidylethanolamine); PG, (phosphatidyl glycerol); PS, (phosphatidylserine).

### Fatty acids and their metabolites

The contents of C20 fatty acids and their metabolites, eicosanoids (eicosapentaenoic acid (EPA), *P* = 0.026, *t*-test, Table 1), arachidonic acid (*P* = 0.025, *t*-test, Table 1), 12-hydroxy-eicosatetraenoic-acid (12-HETE, *P* = 0.009, *t*-test, Table 1), 12*S*-hydroperoxy-5*Z*, 8*Z*, 10*E*, 14*Z*-eicosatetraenoic acid (12(*S*)-HPETE, *P* = 0.016, *t*-test, Table 1), prostaglandin E2 (PGE2, *P* = 0.010 in ESI^+^ mode and *P* = 0.014 in ESI^-^ mode, *t*-test, Table 1), 15-keto-13,14-dihydro-prostaglandin A2 (15-keto-13,14-dihydro-PGA2, *P* = 0.014, *t*-test, Table 1), thromboxane B2 (TXB2, *P* = 0.001, *t*-test, Table 1), were smaller, whereas that of C18 fatty acid metabolite,12,13-dihydroxy-9*Z*-octadecenoic acid (12,13-DHOME) was larger in MTKO mice compared to WT mice (*P* = 0.002, *t*-test, Table 1). Reduced PGE2 levels in muscles contributes to muscle atrophy in aged mice [21].

### Glycolysis/gluconeogenesis and tricarboxylic acid (TCA) cycle metabolites

Levels of L-lactic acid (*P* = 0.006 in ESI^+^ mode and *P* = 0.018 in ESI^-^ mode, *t*-test, Table 1), oxoglutaric acid (*P* = 0.005, *t*-test, Table 1), glycerol 3-phosphate (*P* < 0.001, *t*-test, Table 1), and citric acid (*P* = 0.002, *t*-test, Table 1) were significantly lower in MTKO mice than in WT mice. These results may show differences in activity levels of glycolysis/gluconeogenesis and the TCA cycle between MTKO and WT mice at 50 weeks of age.

### Amino acids and their metabolites

**Figure 2:**
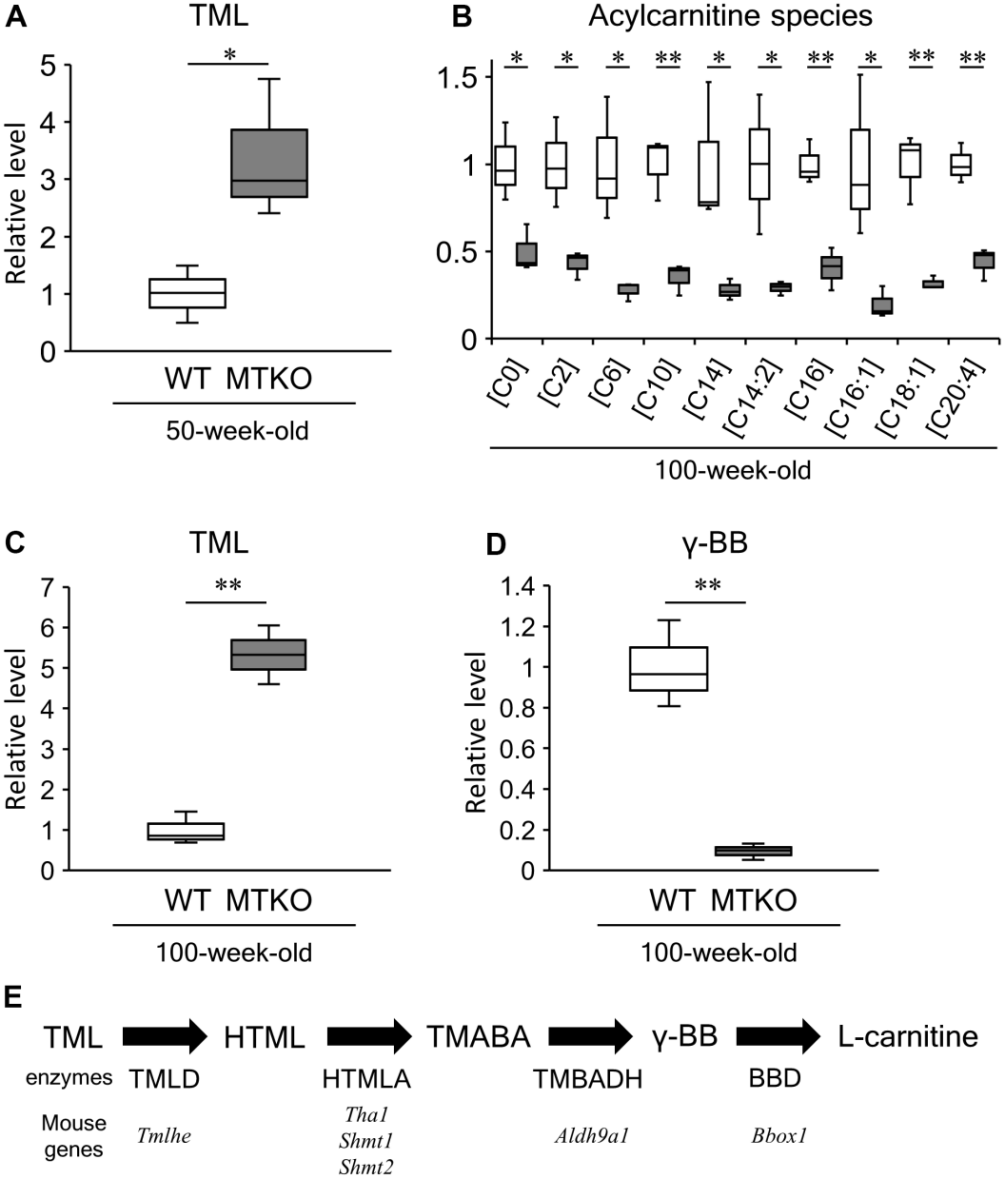
**Levels of acylcarnitine species and carnitine biosynthesis intermediates in plasma of wild-type (WT) mice (white boxes) and MTKO mice (gray boxes).** (**A**) N6,N6,N6-trimethyl-L-lysine (TML) level at 50 weeks of age; (**B**) Levels of L-carnitine [C0] and acylcarnitine species (L-acetylcarnitine [C2], Hexanoylcarnitine [C8], Decanoylcarnitine [C10], Tetradecanoylcarinitne [C14], 3,5-Tetradecadiencarnitine [C14:2], L-Palmitoylcarnitine [C16], 9-Hexadecenoylcarnitine [C16:1], Oleoylcarnitine [C18:1], Arachidonoylcarnitine [C20:4]) at 100 weeks of age; (**C**) TML level at 100 weeks of age; (**D**) Level of γ-butryobetaine (γ-BB), also known as 4-trimethylammoniobutanoic acid, at 100 weeks of age. The box denotes 25th and 75th percentiles; the line within the box denotes the 50th percentile; the whisker denotes standard deviation. (n = 3). *P < 0.05 and **P < 0.01. (**E**) Carnitine biosynthesis pathway. HTML, 3-hydroxyl-N6-trimethyl-L-lysine; TMABA, 4-trimethyl-aminobutyraldehyde; TML dioxygenase, TMLD; HTML aldolase, HTMLA; TMABA dehydrogenase, TMABADH; γ-BB dioxygenase, BBD.

Among amino acids and their metabolites, the levels of taurine (2-aminoethane-1-sulfonic acid) and *N*-lactoylphenylalanine (*P* = 0.038, *t*-test, Table 1) were lower, whereas those of L-phenylalanine (*P* = 0.026, *t*-test, Table 1), L-lysine (*P* = 0.019, *t*-test, Table 1), *N*6*,N*6*,N*6-trimethyl-L-lysine (TML) (*P* = 0.035, *t*-test, Table 1 and Figure 2A), and histidylglutamic acid (*P* = 0.013, *t*-test, Table 1)were significantly higher in the plasma of MTKO mice than in that of WT mice. It has been reported that the plasma level of *N*-lactoylphenylalanine strongly correlates with plasma levels of lactate and L-phenylalanine [22]. Therefore, the low level of L-lactic acid could lead to L-phenylalanine accumulation in the plasma of MTKO mice. TML is an *N*-methylated compound of L-lysine. It is a component of histone proteins and an intermediate that is recognized as the precursor of L-carnitine (Figure 2E). The metabolic ability to L-lysine and TML may be weaker in MTKO mice than in WT mice. Hexadecanedioic acid, which is a long-chain dicarboxylic fatty acid, is converted to hexadecanedioylcarnitine by carnitine palmityltransferase (CPT) and is activated by mitochondrial fractions in the human liver for its degradation by β-oxidation [23]. In the plasma of MTKO mice, the concentration of hexadecanedioic acid (FC = 2.391, *P* = 0.001, *t*-test, Table 1) may be associated with the accumulation of TML as a carnitine precursor.

### Bile acids and bilirubin

The amounts of conjugated primary bile acids, glycocholic acid (*P* < 0.001, *t*-test, Table 1), taurocholic acid (*P* = 0.014, *t*-test, Table 1), and glycochenodeoxycholate-3-sulfate (*P* = 0.005, *t*-test, Table 1) were lower in MTKO mice than in WT mice. The level of taurocholic acid was correlated with different taurine levels in the plasma of both MTKO and WT mice. In contrast, the bilirubin level in the plasma of MTKO mice was markedly higher than that in the plasma of WT mice (*P* = 0.007 in ESI^+^ mode and *P* = 0.004 in ESI^-^ mode, *t*-test, Table 1). In the liver, bilirubin is conjugated with glucuronic acid by glucuronyltransferase. Conjugated bilirubin is then excreted from hepatocytes into the bile canaliculus. The capacity of several conjugation reactions may be limited in the liver of MTKO mice.

### Nucleobases and nucleosides

Among nucleobases and nucleosides, the contents of xanthine (*P* = 0.031, *t*-test, Table 1) and its nucleoside xanthosine (*P* = 0.033 in ESI^+^ mode and *P* = 0.001 in ESI^-^ mode, *t*-test, Table 1) were remarkably lower in MTKO mice than in WT mice. Xanthine oxidoreductase (XOR) generates ROS, such as superoxide radicals and hydrogen peroxide, when it catalyzes the oxidation of hypoxanthine to xanthine, followed by the oxidation of xanthine to uric acid [24]. However, the ROS, which are synthesized in moderate amounts from xanthine with the catalytic activity of XOR, are essential for the appropriate functioning of many physiological pathways, thereby contributing to successful aging [25]. There is no evidence of high levels of ROS in aged MTKO mice. However, excessive lipid peroxidation, an indicator of oxidative stress, was observed in some tissues, including the heart [26], liver [27], and neurons [28] of MTKO mice treated with various anthropogenic stressors. Alterations in mitochondrial homeostasis, including a progressive decrease in ROS scavengers, cause ineffective ROS control and mediate cell stress responses towards age-dependent damage [29]. Due to the deficiency of MT as a ROS scavenger in mitochondria [30], the low level of xanthine in the plasma from MTKO mice might be the result of high utilization of xanthine to address a progressive generation of ROS during the aging process.

### Other compounds

Among phospholipids, levels of lysophospholipid species were lower in the plasma of MTKO mice compared to that of WT mice (all *P* < 0.05, *t*-test, Table 1). Regarding metabolites probably derived from foods, the levels of 4-hydroxybenzaldehyde (*P* = 0.001, *t*-test, Table 1) and indole-3-carboxaldehyde (*P* = 0.001, *t*-test, Table 1) were significantly lower, whereas those of DL-2-aminooctanoic acid (*P* < 0.001, *t*-test, Table 1), dehydrochorismic acid (*P* = 0.012, *t*-test, Table 1), and hydroxystilbamidine (*P* = 0.002, *t*-test, Table 1) were significantly higher in the plasma of MTKO mice than in that of WT mice. These results may show differences in metabolism of the above-mentioned compounds from foods between MTKO and WT mice during a 6 h fast before plasma collection.

### Quantitative comparison of metabolites in the plasma between MTKO and WT 100-week-old male mice

### Identification of metabolites in the plasma

We identified 616 differential metabolites, of which 290 were detected in the positive ion mode and 326 in the negative ion mode in 100-week-old mouse plasma. HCA results of compounds with statistically significant differences between the MTKO and WT male groups are summarized in Table 2. The levels of 42 metabolites were higher, whereas those of 40 were lower in the plasma of MTKO mice than that in WT mice. Interestingly, many of these metabolites are involved in carnitine metabolism.

**Table 2:** HCA data; identified metabolites showing a difference between MTKO and WT male group under ESI^-^ and ESI^+^ scans of 100-week-old mice (*n* = 3).

**No.**	**Compound name**	**Molecular formula**	**RT^a^ [min]**	**Monoisotopic mass**	**Delta (ppm)**	**FC^b^**	***P*-value**	**VIP^c^**
**Positive ion (ESI+)**								
1	Taurine	C2H7NO3S	19.20	125.0147	26	0.257	0.045	1.63
2	Creatine	C4H9N3O2	0.80	131.0695	4	0.358	0.000	1.98
3	*N*-Acetylvaline	C7H13NO3	3.82	159.0895	4	2.309	0.022	1.74
4	L-Phenylalanine	C9H11NO2	0.83	165.0790	4	3.391	0.002	1.91
5	Indole-3-propionic acid	C11H11NO2	7.31	189.0790	4	2.632	0.018	1.77
6	Indolelactic acid	C11H11NO3	6.08	205.0739	4	2.406	0.044	1.64
7	Hippuric acid	C9H9NO3	4.39	179.0582	4	3.670	0.013	1.80
8	*N*6,*N*6,*N*6-Trimethyl-L-lysine	C9H20N2O2	0.74	188.1525	4	5.326	0.001	1.94
9	4-Trimethylammoniobutanoic acid	C7H15NO2	0.80	145.1103	3	0.093	0.002	1.91
10	L-Carnitine	C7H15NO3	0.81	161.1052	3	0.500	0.029	1.70
11	L-Acetylcarnitine	C9H17NO4	0.81	203.1158	4	0.430	0.022	1.74
12	Hexanoylcarnitine	C13H25NO4	5.28	259.1784	3	0.277	0.025	1.72
13	Decanoylcarnitine	C17H33NO4	8.44	315.2410	4	0.351	0.005	1.87
14	3, 5-Tetradecadiencarnitine	C21H37NO4	9.62	367.2723	3	0.292	0.037	1.66
15	Tetradecanoylcarnitine	C21H41NO4	10.85	371.3036	3	0.279	0.039	1.66
16	9-Hexadecenoylcarnitine	C23H43NO4	11.22	397.3192	3	0.199	0.043	1.64
17	L-Palmitoylcarnitine	C23H45NO4	11.98	399.3349	3	0.405	0.004	1.88
18	Oleoylcarnitine	C25H47NO4	12.26	425.3505	3	0.319	0.004	1.88
19	Arachidonoylcarnitine	C27H45NO4	11.59	447.3349	3	0.439	0.003	1.90
20	Eicosapentaenoyl Ethanolamide	C22H35NO2	15.86	345.2668	3	0.385	0.035	1.67
21	*N*-Oleoyl tyrosine	C27H43NO4	11.03	445.3192	3	0.426	0.030	1.70
22	Succinyladenosine	C14H17N5O8	0.89	383.1077	14	0.200	0.023	1.74
23	LysoPC(16:0/0:0)	C24H50NO7P	11.79	495.3325	1	0.396	0.025	1.72
24	LysoPE(0:0/20:3(5*Z*,8*Z*,11*Z*))	C25H46NO7P	12.62	503.3012	2	0.459	0.048	1.62
25	LysoPC(18:3(6Z,9Z,12Z)/0:0)	C26H48NO7P	11.46	517.3168	4	2.221	0.006	1.86
26	LysoPC(18:2(9Z,12Z))	C26H50NO7P	12.87	519.3325	2	2.205	0.034	1.68
27	LysoPC(20:0)	C28H58NO7P	15.07	551.3951	3	0.358	0.021	1.75
28	LysoPC(22:2(13Z,16Z)/0:0)	C30H58NO7P	14.98	575.3951	3	0.408	0.043	1.64
29	PC(18:3(6Z,9Z,12Z)/P-16:0)	C42H78NO7P	15.87	739.5516	2	2.764	0.019	1.76
30	PC(16:0/22:6(4Z,7Z,10Z,13Z,16Z,19Z))	C46H80NO8P	16.25	805.5622	0	8.493	0.015	1.78
31	PC(18:0/20:3(5Z,8Z,11Z))	C46H86NO8P	19.12	811.6091	2	3.231	0.001	1.93
32	PC(20:5(5Z,8Z,11Z,14Z,17Z)/ 20:4(5Z,8Z,11Z,14Z))	C48H78NO8P	16.25	827.5465	2	10.691	0.004	1.88
33	SM(d18:1/24:1(15Z))	C47H93N2O6P	19.13	812.6771	0	19.326	0.026	1.72
34	Proline betaine	C7H13NO2	0.80	143.0946	4	2.115	0.004	1.88
35	DL-2-Aminooctanoic acid	C8H17NO2	0.82	159.1259	3	3.365	0.012	1.80
**Negative ion (ESI-)**								
36	3-Dehydrocarnitine	C7H13NO3	3.83	159.0895	9	2.499	0.004	1.73
37	Ketoleucine	C6H10O3	3.76	130.0630	12	2.144	0.010	1.68
38	3-Hydroxymethylglutaric acid	C6H10O5	0.88	162.0528	9	0.444	0.007	1.70
39	3-Phenyllactic acid	C9H10O3	5.12	166.0630	9	2.694	0.039	1.52
40	Kynurenic acid	C10H7NO3	3.70	189.0426	7	2.612	0.018	1.62
41	cyclic 6-Hydroxymelatonin	C13H14N2O3	6.04	246.1004	4	4.580	0.008	1.69
42	gamma-Glutamylvaline	C10H18N2O5	3.42	246.1216	3	3.216	0.000	1.80
43	Hippuric acid	C9H9NO3	4.40	179.0582	7	3.729	0.011	1.67
44	2-Methylhippuric acid	C10H11NO3	4.87	193.0739	7	2.430	0.012	1.66
45	Suberylglycine	C10H17NO5	4.21	231.1107	4	3.816	0.003	1.74
46	Palmitoylglycine	C18H35NO3	15.17	313.2617	2	0.457	0.015	1.64
47	Oleoyl glycine	C20H37NO3	15.55	339.2773	3	0.440	0.029	1.56
48	Palmitic acid	C16H32O2	16.94	256.2402	4	0.350	0.005	1.72
49	Oleic acid	C18H34O2	17.14	282.2559	3	0.491	0.012	1.66
50	Sebacic acid	C10H18O4	4.98	202.1205	6	2.010	0.032	1.55
51	Dodecanedioic acid	C12H22O4	8.34	230.1518	5	2.378	0.004	1.73
52	Tetradecanedioic acid	C14H26O4	8.42	258.1831	3	4.188	0.003	1.74
53	3-Hydroxytetradecanedioic acid	C14H26O5	6.35	274.1780	3	8.523	0.005	1.72
54	3-Oxododecanoic acid	C12H22O3	10.37	214.1569	5	0.483	0.032	1.55
55	3-Hydroxydodecanoic acid	C12H24O3	10.43	216.1725	5	3.573	0.008	1.69
56	Myristic acid	C14H28O2	15.57	228.2089	5	0.472	0.043	1.51
57	2-Hydroxymyristic acid	C14H28O3	11.55	244.2038	4	5.260	0.011	1.66
58	Ricinoleic acid	C18H34O3	15.26	298.2508	3	0.261	0.033	1.55
59	Mead acid	C20H34O2	16.63	306.2559	3	0.410	0.020	1.61
60	13-L-Hydroperoxylinoleic acid	C18H32O4	12.66	312.2301	2	2.571	0.024	1.59
61	Docosapentaenoic acid (22n-3)	C22H34O2	16.27	330.2559	3	0.463	0.032	1.55
62	Docosatetraenoic acid (22n-6)	C22H36O2	16.97	332.2715	3	0.362	0.016	1.64
63	12(S)-HPETE	C20H32O4	10.76	336.2301	3	2.631	0.037	1.53
64	N-Stearoyltaurine	C20H41NO4S	17.02	391.2756	3	6.329	0.020	1.61
65	ADP	C10H15N5O10P2	14.02	427.0294	18	0.494	0.030	1.56
66	5alpha-Tetrahydrocortisol	C21H34O5	16.30	366.2406	25	0.409	0.026	1.58
67	Androsterone glucuronide	C25H38O8	17.16	466.2567	14	0.402	0.000	1.81
68	LysoPE(20:4(5Z,8Z,11Z,14Z)/0:0)	C25H44NO7P	19.22	501.2855	3	2.287	0.027	1.58
69	LysoPE(0:0/20:2(11Z,14Z))	C25H48NO7P	13.43	505.3168	4	0.499	0.027	1.57
70	LysoPE(0:0/20:0)	C25H52NO7P	15.85	509.3481	4	0.386	0.002	1.76
71	LysoPE(0:0/22:6(4Z,7Z,10Z,13Z,16Z,19Z))	C27H44NO7P	11.88	525.2855	3	0.471	0.034	1.54
72	LysoPS(18:0(9Z)/0:0)	C24H48NO9P	19.35	525.3067	3	0.485	0.021	1.61
73	LysoPE(22:2(13Z,16Z)/0:0)	C27H52NO7P	14.90	533.3481	3	0.129	0.009	1.68
74	LysoPI(0:0/18:0)	C27H53O12P	11.34	600.3275	3	28.370	0.011	1.66
75	LysoPI(20:4(5Z,8Z,11Z,14Z)/0:0)	C29H49O12P	15.22	620.2962	3	26.399	0.021	1.60
76	PG(16:1(9Z)/18:0)	C40H77O10P	19.28	748.5254	2	5.073	0.003	1.74
77	PS(20:2(11Z,14Z)/15:0)	C41H76NO10P	19.32	773.5207	0	0.421	0.003	1.75
78	Protocatechuic acid	C7H6O4	4.00	154.0266	9	15.130	0.005	1.72
79	(S)-Oleuropeic acid	C10H16O3	8.16	184.1099	7	3.758	0.030	1.56
80	Caffeic acid 3-O-sulfate	C9H8O7S	4.27	259.9991	3	4.317	0.004	1.73
81	Ferulic acid 4-O-sulfate	C10H10O7S	4.30	274.0147	2	9.682	0.000	1.80
82	2-Phenylethyl beta-D-glucopyranoside	C14H20O6	5.18	284.1260	3	9.735	0.034	1.54

RT^a^, retention time; FC^b^, fold change in the comparison (average in MTKO mice/WT mice); VIP^c^, variable importance in the projection scores derived from discriminant analysis. The metabolites were filtered and confirmed by combining the results of the VIP values (VIP > 1.5), FC values (FC > 2.0), and t-test (*P* < 0.05).

Abbreviations: PC, (phosphatidylcholine); SM, (sphingomyelin); 12(S)-HPETE, (12S-hydroperoxy-5Z, 8Z, 10E, 14Z-eicosatetraenoic acid); ADP, (adenosine diphosphate); PE, (phosphatidylethanolamine); PS, (phosphatidylserine); PI, (phosphatidylinositol); PG, (phosphatidyl glycerol).

### Carnitine metabolites

Levels of L-carnitine ((*R*)-3-hydroxy-4-(trimethylammonio) butyrate) and acylcarnitine species with different acyl chains were significantly lower in MTKO mice than in WT mice (all *P* < 0.05, *t*-test, Table 2 and Figure 2B). In contrast, TML levels in the plasma of MTKO mice were 5.33-fold greater than that in the plasma of WT mice (*P* = 0.001, *t*-test, Table 2) (Figure 2C). 4-trimethylammoniobutanoic acid, also known as γ-butryobetaine (γ-BB), is a metabolic intermediate in L-carnitine biosynthesis (Figure 2E), and its quantity in MTKO mice was less than one-tenth that in WT mice (*P* = 0.002, *t*-test, Table 2) (Figure 2D). Network analysis indicated that metabolites related to carnitine synthesis differed significantly between MTKO and WT mice (*P* = 0.0388, obtained from analysis of MBRole, Table 3 and Supplementary Figure 5). The level of 3-dehydrocarnitine, which is an intermediate in carnitine degradation, was 2.5-fold higher in MTKO mice than in WT mice (*P* = 0.004, *t*-test, Table 2).

**Table 3:** Pathway enrichment analysis of identified metabolites showing a difference between MTKO and WT male group under ESI^+^ scan of 100-week-old mice.

**Pathway name**	**Total**	**Hits**	**Expect**	***P* value**	**Holm *P***	**FDR**
Carnitine Synthesis	22	3	0.773	0.0388	1.0	1.0
Beta Oxidation of Very Long Chain Fatty Acids	17	2	0.598	0.117	1.0	1.0
Oxidation of Branched Chain Fatty Acids	26	2	0.914	0.231	1.0	1.0
Taurine and Hypotaurine Metabolism	12	1	0.422	0.351	1.0	1.0
Fatty acid Metabolism	43	2	1.51	0.453	1.0	1.0
Mitochondrial Beta-Oxidation of Short Chain Saturated Fatty Acids	27	1	0.949	0.624	1.0	1.0
Phenylalanine and Tyrosine Metabolism	28	1	0.984	0.638	1.0	1.0
Mitochondrial Beta-Oxidation of Long Chain Saturated Fatty Acids	28	1	0.984	0.638	1.0	1.0
Phospholipid Biosynthesis	29	1	1.02	0.651	1.0	1.0
Arginine and Proline Metabolism	53	1	1.86	0.857	1.0	1.0
Glycine and Serine Metabolism	59	1	2.07	0.886	1.0	1.0
Bile Acid Biosynthesis	65	1	2.29	0.91	1.0	1.0

A *P*-value was obtained from analysis of MBRole. Total, the total number of metabolite in the pathway dataset; Expect, the expectation value; Hits, the number of metabolites in the experimental dataset matching the pathway dataset; Holm *P*, the *P* value adjusted by Holm-Bonferroni method; FDR, the *P* value adjusted using False Discovery Rate.

### Aliphatic dicarboxylic acids and fatty acid metabolites

The levels of sebacic acid, dodecanedioic acid, tetradecanedioic acid, and 3-hydroxytetradecanedioic acid, which are aliphatic dicarboxylic acids, were higher in the plasma of MTKO mice than in the plasma of WT mice (*P* = 0.032, 0.004, 0.003, and 0.005, respectively, *t*-test, Table 2). In humans, levels of sebacic acid are increased in the urine of patients with carnitine-acylcarnitine translocase deficiency, which is an inborn error of metabolism [31]. Dodecanedioic acid is a marker of hepatic CPT Iα-deficiency [32]. 3-hydroxytetradecanedioic acid is detected in the urine of patients with thanatophoric dysplasia type I with fibroblast growth factor receptor 3 gene mutation (S249C) due to incomplete fatty acid metabolism [33].

Hippuric acid, 2-methylhippuric acid, and suberylglycine are acyl glycines, which are minor fatty acid metabolites. The levels of these acyl glycines are increased in disorders associated with mitochondrial fatty acid β-oxidation. Thus, in the plasma of MTKO mice, higher levels of these minor fatty acid metabolites (all *P* < 0.05, *t*-test, Table 2) and the markers of fatty acid metabolism disorder can be ascribed to the lower levels of carnitine compounds. Levels of acyl glycines, palmitoylglycine, and oleoyl glycine were lower in the plasma of MTKO mice compared to that of WT mice (*P* = 0.015 and 0.029, respectively, *t*-test, Table 2). This may be because the plasma levels of palmitic acid and oleic acid were also lower in MTKO mice than in WT mice (*P* = 0.005 and 0.012, respectively, *t*-test, Table 2). The capacity of fatty acid catabolism and fatty acid biosynthesis in MTKO mice may be weaker than that in WT mice.

Other minor fatty acid metabolites were also identified. The level of 3-oxododecanoic acid in MTKO mice was lower (*P* = 0.032, *t*-test, Table 2), but that of its hydroxide, 3-hydroxydodecanoic acid, was higher in MTKO mice than in WT mice (*P* = 0.008, *t*-test, Table 2). This tendency was similar to that of myristic acid (*P* = 0.043, *t*-test, Table 2) and its hydroxide, 2-hydroxymyristic acid (*P* = 0.011, *t*-test, Table 2). In humans, 3-oxododecanoic acid is involved in fatty acid biosynthesis, and 3-oxododecanoic acid can be converted into 3-hydroxydodecanoic acid. 3-hydroxydodecanoic acid is a fatty acid associated with fatty acid metabolic disorders, such as medium-chain acyl CoA dehydrogenase deficiency, which shows low plasma and tissue carnitine levels [34, 35]. Levels of ricinoleic acid and mead acid, which are unsaturated n-9 fatty acid metabolites, were lower in MTKO mice (*P* = 0.033 and 0.020, respectively, *t*-test, Table 2). Levels of 13-L-hydroperoxylinoleic acid (13(*S*)-HPODE) and 12(*S*)-HPETE were greater in MTKO mice than those in WT mice (*P* = 0.024 and 0.037, respectively, *t*-test, Table 2). Further, levels of very long chain fatty acids, docosapentaenoic acid (22n-3), and docosatetraenoic acid (22n-6) were lower in MTKO mice than in WT mice (*P* = 0.032 and 0.016, respectively, *t*-test, Table 2). The levels of *N*-oleoyl-tyrosine and eicosapentaenoyl ethanolamide (EPEA) were lower in MTKO mice than in WT mice (*P* = 0.030 and 0.035, respectively, *t*-test, Table 2), although *N*-stearoyltaurine levels in MTKO mice were 6.3-fold higher than that in WT mice (*P* = 0.020, *t*-test, Table 2).

### Phospholipids

Phospholipid species levels were higher, whereas lysophospholipid species were lower in the plasma of MTKO mice compared to that in the plasma of WT mice. As an exception, the levels of lysophosphatidylinositols, lysoPI (0:0/18:0), and lysoPI (20:4 (5*Z*, 8*Z*, 11*Z*, 14*Z*)/0:0) were significantly higher (FC > 26) in MTKO mice than in WT mice (*P* = 0.011 and 0.021, respectively, *t*-test, Table 2).

### Metabolites of amino acids and other compounds

Other than carnitine species, various amino acid metabolites were also identified. The levels of taurine and creatine (2-(*N*-methylcarbamimidamido) acetic acid) in the plasma of MTKO mice were lower than those in the plasma of WT mice (*P* = 0.045 and *P* < 0.001, respectively, *t*-test, Table 2). Both are mainly biosynthesized in the liver and are widely distributed in various tissues, especially in the skeletal muscle, heart, brain, liver, kidney, and retina. Levels of valine metabolites (*N*-acetylvaline and γ-glutamylvaline (*P* = 0.022 and *P* < 0.001, respectively, *t*-test, Table 2)), L-phenylalanine (*P* = 0.002, *t*-test, Table 2) and its metabolite (3-phenyllactic acid, *P* = 0.039, *t*-test, Table 2), tryptophan metabolites (indole-3-propionic acid, indolelactic acid, kynurenic acid, and cyclic 6-hydroxymelatonin (*P* = 0.018, 0.044, 0.018, and 0.008, respectively, *t*-test, Table 2)), and ketoleucine (*P* = 0.010, *t*-test, Table 2) were higher in MTKO mice than in WT mice. Among ribonucleotide metabolites, levels of succinyladenosine, a precursor of AMP and ADP, were lower in MTKO mice than in WT mice (*P* = 0.023, *t*-test, Table 2). Among steroid metabolites, levels of 5α-tetrahydrocortisol and androsterone glucuronide in MTKO mice were lower than that in WT mice (*P* = 0.026 and *P* < 0.001, respectively, *t*-test, Table 2). Levels of some metabolites, probably derived from plant foods, such as proline betaine (*P* = 0.004, *t*-test, Table 2), protocatechuic acid (*P* = 0.005, *t*-test, Table 2), (*S*)-oleuropeic acid (*P* = 0.030, *t*-test, Table 2), caffeic acid 3-O-sulfate (*P* = 0.004, *t*-test, Table 2), ferulic acid 4-O-sulfate (*P* < 0.001, *t*-test, Table 2), and 2-phenylethyl β-D-glucopyranoside (*P* = 0.034, *t*-test, Table 2), and those probably derived from animal foods, such as DL-2-Aminooctanoic acid (*P* = 0.012, *t*-test, Table 2), were significantly higher in the plasma of MTKO mice compared to that in WT mice. The results may show differences in metabolism of these compounds between MTKO and WT mice during a 6 h fast before plasma collection.

### Common features and properties of metabolites in plasma among 50-week- and 100-week-old mice

### Compounds found in smaller quantities in the plasma of male MTKO mice compared to that of male WT mice

Levels of the common compound taurine were lower in the plasma of male MTKO mice compared to that of male WT mice at both 50 and 100 weeks of age (Tables 1, 2). Taurine has several functions, such as conjugation with bile acids, antioxidants, osmolytes, membrane stabilizers, and as a modulator of calcium signaling [36–38]. It protects the cardiovascular system [39], skeletal muscle [40, 41], retina [42], and central nervous system [43] against pathology and disease [44]. Taurine depletion shortens the lifespan of mice [45, 46]. Since rodents, unlike cats and humans, exhibit considerable biosynthetic capacity for taurine [47], *MT* gene deficiency would have a significant influence on the biosynthesis pathway and plasma levels of taurine in mice. Taurine levels in the plasma of MTKO mice were approximately one-third of that in WT mice at 50 weeks of age, and this level decreased in 100-week-old WT mice to approximately one-fourth of the previously observed level. This decrease in relative values with age may lead to muscular depression, and disorders of the cardiovascular system, the eye, and central nervous system in MTKO mice, and may finally reduce lifespan. Unlike taurine, creatine levels were lower in 100-week-old MTKO mice compared to WT mice. Creatine is mainly synthesized in the liver and has potential beneficial roles that are nearly as effective as taurine in cardiovascular function, muscle mass, cognitive function, and lifespan [38, 48]. The low levels may be a result of accelerated aging of the liver caused by MT deficiency.

### Compounds found in larger quantities in the plasma of male MTKO mice versus that of WT mice

The common metabolites that were found to be consistently higher in the plasma of MTKO mice compared to that of WT mice at 50 and 100 weeks of age were L-phenylalanine, TML, and DL-2-aminooctanoic acid (Tables 1, 2). Phenylketonuria (PKU) is an inborn error of metabolism that results from a deficiency in phenylalanine hydroxylase (PAH), the enzyme catalyzing the conversion of phenylalanine to tyrosine. PKU results in severe hyperphenylalaninemia. The enzyme activity of PAH may decrease with age in the MTKO mice. However, similar to that of a mouse model of mild hyperphenylalaninemia (*Pah^enu1^* variant) [49], the level of phenylalanine in the plasma of MTKO mice may have little effect on its lifespan. DL-2-aminooctanoic acid is an α-amino fatty acid derived from food. The higher level of DL-2-aminoctanoic acid in the plasma of MTKO mice compared to WT mice may be caused by the low metabolic activity of fatty acids.

### Influences of MT deficiency on the carnitine biosynthesis pathway

In animals, L-carnitine is biosynthesized from TML in four enzyme-catalyzed steps, as summarized in Figure 2E [50, 51]. The first enzyme, TML dioxygenase (TMLD), catalyzes the hydroxylation of TML to produce 3-hydroxy-TML (HTML). TMLD is encoded by the TML hydroxylase, epsilon (*Tmlhe*) gene in mice. In the second step, HTML aldolase (HTMLA) catalyzes the aldolytic cleavage of HTML into 4-*N*-trimethylaminobutyraldehyde (TMABA) and glycine. In mice, it is currently thought that threonine aldolase 1 (THA1, encoded by *Tha1*), and serine hydroxymethyltransferase (SHMT, encoded by *Shmt*) possess HTMLA activity. The third enzyme, TMABA dehydrogenase (TMABADH), catalyzes the dehydrogenation of TMABA into γ-BB. TMABADH is encoded by the aldehyde dehydrogenase 9 subfamily A1 (*Aldh9a1*) gene in mice. In the final step, γ-BB dioxygenase (BBD) catalyzes the hydroxylation of γ-BB into L-carnitine. BBD is encoded by the γ-BB, 2-oxoglutarate dioxygenase 1 (*Bbox1*) gene in mice. The highest TMLD activity is found in the kidney but is also present in the liver, skeletal muscle, heart, and brain in rats and humans [52–54]. The highest enzyme activities for subsequent processes converting HTML to L-carnitine are seen in the liver of rodents. OCTN2, which is encoded by the *Slc22a5* gene, is an organic cation/carnitine transporter that controls the storage of L-carnitine [55].

The relative level of TML in the plasma of MTKO mice was 3.38-fold of that recorded in WT mice at 50 weeks of age (Table 1 and Figure 2A), and then increased to 5.33-fold of that in WT mice at 100 weeks of age (Table 2 and Figure 2C). Levels of γ-BB, L-carnitine, and acylcarnitines recorded in MTKO mice were lower than those observed in 100-week-old WT mice, unlike 50-week-old mice. These results show that the TML level in the plasma of MTKO mice was consistently higher than that observed in the plasma of WT mice, and that the lower levels of γ-BB and L-carnitine in MTKO mice compared to WT mice are dependent on aging.

**Figure 3:**
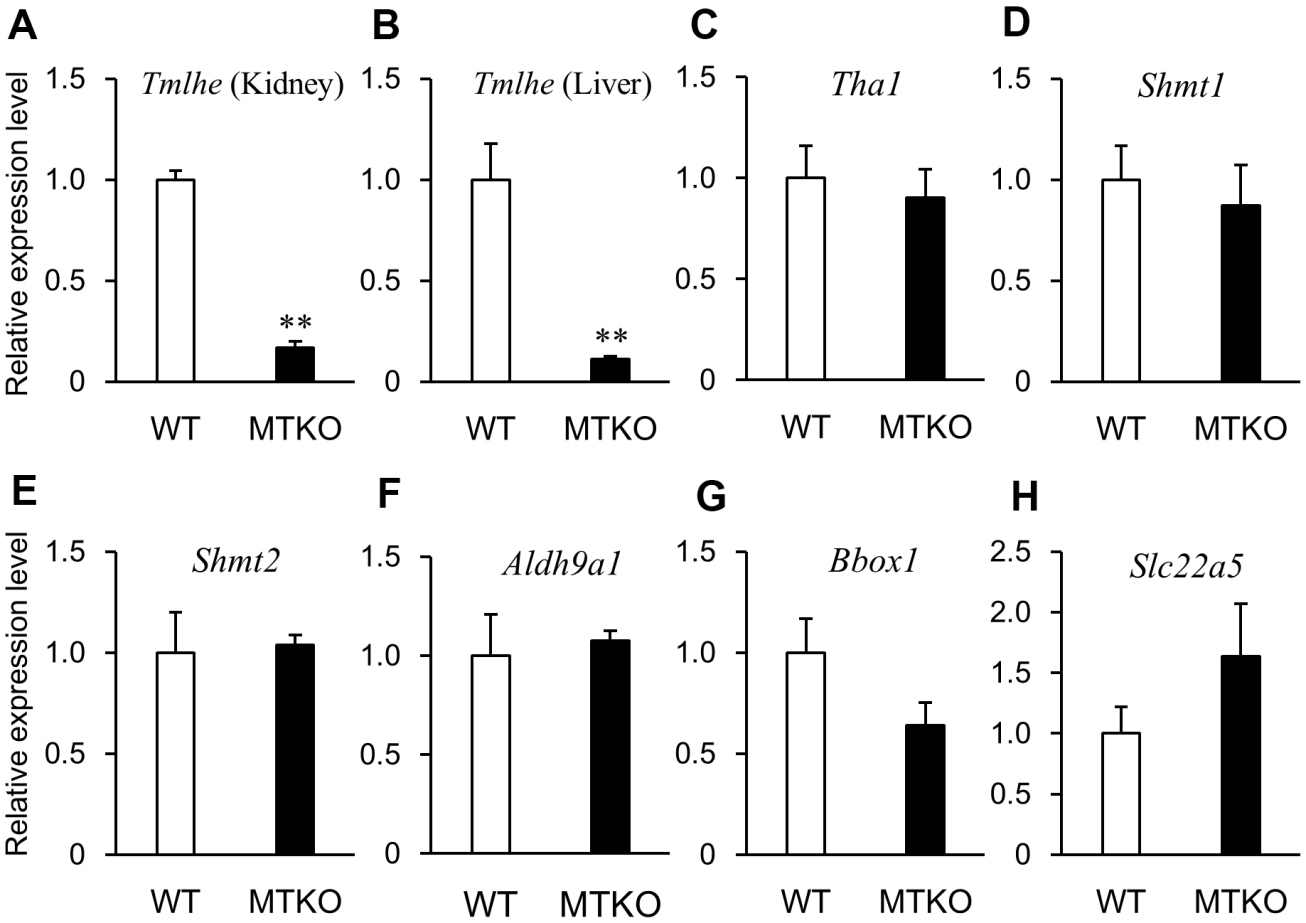
**mRNA expression levels of enzymes associated with carnitine biosynthesis and carnitine transporter in wild-type (WT) mice (white columns) and MTKO mice (black columns) at 50 weeks of age.** (**A**) *Tmlhe* gene in kidney; (**B**) *Tmlhe* gene, (**C**) *Tha1* gene, (**D**) *Shmt1* gene, (**E**) *Shmt2* gene, (**F**) *Aldh9a1* gene, (**G**) *Bbox1* gene, and (**H**) *Slc22a5* gene in liver. The data are expressed as ratios to the mean values in the WT group. Values are presented as the mean ± SE (*n* = 3). *P < 0.05 and **P < 0.01.

**Figure 4:**
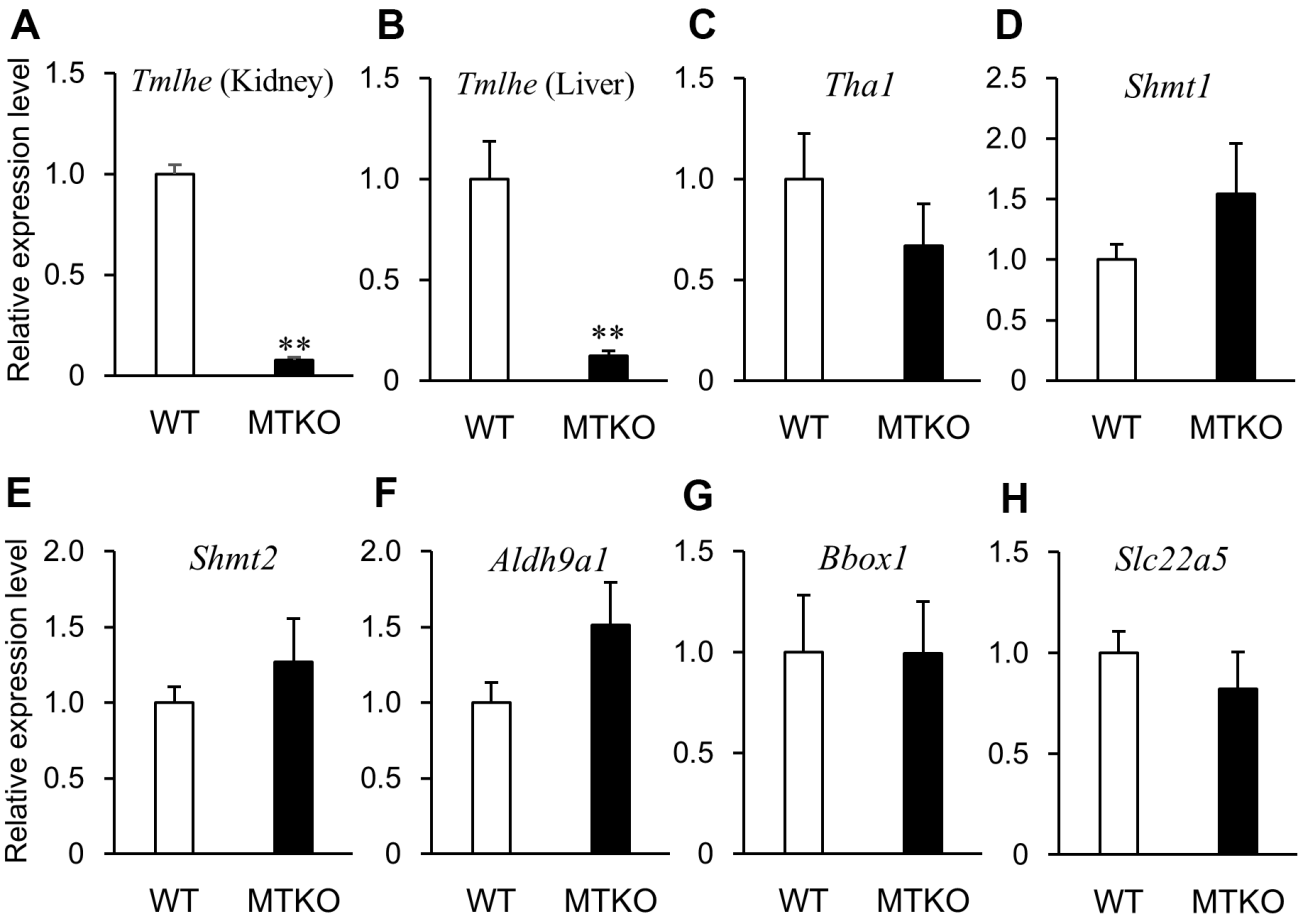
**mRNA expression levels of enzymes associated with carnitine biosynthesis and carnitine transporter in wild-type (WT) mice (white columns) and MTKO mice (black columns) at 100 weeks of age.** (**A**) *Tmlhe* gene in kidney; (**B**) *Tmlhe* gene, (**C**) *Tha1* gene, (**D**) *Shmt1* gene, (**E**) *Shmt2* gene, (**F**) *Aldh9a1* gene, (**G**) *Bbox1* gene, and (**H**) *Slc22a5* gene in liver. The data are expressed as ratios to the mean values in the WT group. Values are presented as the mean ± SE (*n* = 3). *P < 0.05 and **P < 0.01.

In order to investigate why levels of carnitine-related metabolites in plasma differed between MTKO and WT mice and if there was a time-dependent change in their levels, we measured the mRNA expression levels of enzymes associated with the carnitine biosynthesis pathway and carnitine transporter in the liver and kidney of 50-week- and 100-week-old male mice. Quantitative reverse transcription PCR (RT-PCR) analysis showed that the mRNA level of *Tmlhe* in MTKO mice was about one-tenth of that in WT mice at both middle and advanced age in the kidney (Figures 3A, 4A) and liver (Figures 3B, 4B). There were statistically non-significant differences in the mRNA levels of other enzymes (*Tha1* (Figures 3C, 4C), *Shmt1* (Figures 3D, 4D), *Shmt2* (Figures 3E, 4E), *Aldh9a1* (Figures 3F, 4F), *Bbox1* (Figures 3G, 4G)), and transporter (*Slc22a5* (Figures 3H, 4H)) in the liver of MTKO and WT mice. Therefore, the higher level of TML in the plasma of MTKO mice at 50 weeks and 100 weeks of age would be caused by the constant lower expression level of *Tmlhe* in the kidney and liver of MTKO mice compared to WT mice. The low TMLD activity of the kidney and liver in MTKO mice may decrease the metabolism from TML to HTML at middle age, suggesting accumulation of TML in plasma, and eventually decrease the biosynthesis of γ-BB and L-carnitine at advanced ages.

## Discussions

In humans, MT levels show an age-dependent increase in the liver and kidney [56] and increase in the mild old age, and decline in very old age in lymphocytes [57]. However, there are individual differences in the expression levels of MTs [56] and polymorphisms in MT loci [17]. Since high MT expression could protect organs and cultured cells from oxidative stress-induced senescence in mice and humans [58–61], the amount of MT protein in tissues is critical for the maintenance of organ function and longevity. However, MTs are inducible genes with lower basal expression under mild stressful conditions. Hence, the comparison of aging patterns between MTKO mice and WT mice in daily life would provide a basis for understanding longevity at the basal level of MT. In order to investigate how MT genes deficiency accelerates the aging of mice, we performed a metabolomic analysis of plasma in middle-aged 50-week-old mice and aged 100-week-old mice using LC-TOF-MS.

In this study, we found that various metabolites associated with L-carnitine and fatty acid metabolism were extracted by comparative metabolomic analyses of the plasma in MTKO and WT mice. L-carnitine is essential for the transfer of long-chain fatty acids across the inner mitochondrial membrane for the subsequent β-oxidation of fatty acids [62]. Mitochondrial dysfunction is closely associated with aging [63]. There is an age-related decrease in L-carnitine levels in the brain and plasma of rats, and age-associated mitochondrial decay can be reversed in older rats by feeding them acetylcarnitine [64]. The carnitine shuttle in mitochondria is associated with the lifespan of a *Drosophila* model of amyotrophic lateral sclerosis based on neuronal or glial expression of human TDP-43, which exhibits dyslipidemia [65]. Based on our results, higher TML levels in the plasma of MTKO mice compared to that of WT mice at 50 weeks of age evoked potential hypometabolism of TML in MTKO mice, thereby leading to lower L-carnitine levels in MTKO mice at 100 weeks of age; this was suggested as the cause of higher levels of minor fatty acid metabolites in the plasma of aged MTKO mice than in the plasma of aged WT mice. The fatty acid metabolic disorder in MTKO mice may have led to high-fat diet-induced fatty liver and obesity [66] and a shortened lifespan [20]. Carnitine protects from oxidative stress and extends the lifespan of *C. elegans* [67]. Older MTKO mice with low L-carnitine plasma levels may be vulnerable to oxidative stress enhanced by the deficiency of MT, as MT is an antioxidant. A previous study found that hepatic L-carnitine levels in MTKO mice were significantly lower than those in WT mice, and the L-carnitine level in MTKO mice fed a high-fat diet decreased with an increase in restraint stress intensity; however, the stress had no effect on L-carnitine levels in WT mice [68]. MT proteins may prevent decreasing L-carnitine levels induced by exposure to constant stress and aging. Cancer patients may present with carnitine deficiency [69], and L-carnitine prevents diethylnitrosamine (DENA)-induced hepatic carcinogenesis in rats [70]. In addition, MTKO mice showed a relatively high incidence of macroscopic tumor masses in the liver [71], suggesting that MT proteins prevent liver carcinogenesis by ensuring carnitine synthesis. The levels of L-carnitine and carnitine biosynthesis intermediates are very similar in rats and mice, although large differences in enzyme activities and kinetics of the enzymes involved in carnitine biosynthesis were found between rats and mice [72]. TML administered to rats was almost completely converted to L-carnitine [73]. We demonstrated that plasma TML levels in MTKO mice were significantly higher than levels observed in WT mice, and *Tmlhe* mRNA levels in the kidney and liver in MTKO mice was approximately 10-fold lower than those in WT mice liver and kidney, even at 50 weeks of age, suggesting that the lower TMLD activity in MTKO mice compared to WT mice has an enormous influence on the conversion from TML to HTML, followed by carnitine biosynthesis.

L-carnitine levels in the body often decrease with age [74, 75]. However, mice can also obtain L-carnitine from a daily diet, suggesting that the lower level of L-carnitine and acylcarnitine species in the plasma of MTKO mice compared to plasma of WT mice at 100 weeks of age depends on the low expression level of *Tmlhe* in MTKO mice. Therefore, the effect of *MT* gene deficiency on decreased L-carnitine storage becomes apparent during aging. In fact, the levels of γ-BB, the last intermediate of carnitine biosynthesis, and those of L-carnitine in the plasma of MTKO mice were considerably lower than those in the plasma of WT mice at 100 weeks of age.

The human ortholog of mouse threonine aldolase gene *Tha1*, also known as *Gly1*, which catalyzes the conversion of HTML to TMABA, is a pseudogene [76]. In addition, administration of TML leads to increased excretion of TML in humans [52]. For these reasons, TML metabolism is thought to be weaker in humans than in rodents [50]. Therefore, the role of MT proteins may be more important for carnitine biosynthesis in humans than in rodents, and high levels of TML in plasma may be a biomarker of accelerated aging caused by MT protein dysfunction.

The enzyme activity of rat TMLHE is inhibited by soft metal ions, including cadmium, zinc, and sulfhydryl reagents [53]. MT proteins act as chelators for cadmium and zinc and react with sulfhydryl reagents [77], suggesting that MT proteins protect not only the expression of *Tmlhe* mRNA but also THLHE enzyme activity. This study revealed the metabolic abnormalities of carnitine biosynthesis in MTKO mice, which could trigger lifespan shortening in mice. However, further studies are required to determine why *Tmlhe* mRNA expression was suppressed in the liver and kidney of MTKO mice.

Although we performed sufficient statistical analyses on metabolomics results, it is important for us to note the small sample sizes of MTKO and WT mice at middle age and advanced age in this study. Our sample sizes were smaller due to the cost of animal studies, as well as the humane use of animals in scientific experiments. Therefore, our findings should be interpreted with caution and viewed as results in hypothesis-generating research. Nevertheless, metabolomics analysis of plasma revealed the metabolic abnormalities of carnitine biosynthesis in aged MTKO mice. These abnormalities were attributed to the lower expression of *Tmlhe* mRNA in the kidney and liver of MTKO mice compared to WT mice. This finding may have a profound impact on aging research, as there are few studies depicting the downregulation of the *Tmlhe* gene. Further research is required to elucidate the mechanism underlying downregulation of the *Tmlhe* gene by *MT* gene deficiency. Moreover, metabolites other than carnitine-related compounds deserve further investigation.

## MATERIALS AND METHODS

### Animals and treatment

MTKO mice (129S7/SvEvBrd-Mt1tm1Bri Mt2tm1Bri/J) and WT mice (129S1/SvImJ) were purchased from The Jackson Laboratory (Bar Harbor, ME, USA). These mice were maintained under specific pathogen-free conditions and mated only with the 129/Sv mouse strain to maintain the genetic background. The mice were housed in a temperature-controlled room at 22° C with 55 ± 5% humidity under a 12 h light/dark cycle, fed a chow diet (Oriental Yeast, Tokyo, Japan), and provided with drinking water ad libitum. The study was conducted according to the guidelines of the Japanese Ministry of Education, Culture, Sports, Science, and Technology, and approved by the Ethics Committee of Tokushima Bunri University (protocol code #16-1 and date of approval; 20th April, 2018).

### Sample preparation

Before blood sampling, the mice were fasted for 6 h to reduce the immediate effects of dietary intake on the plasma constituents. The animals were sacrificed using isoflurane (FUJIFILM Wako Pure Chemical, Osaka, Japan), and each organ was removed. Whole blood was collected in a 1.5 mL tube with 60 IU sodium heparin (Nacalai Tesque, Kyoto, Japan) in 25 μL saline, and was centrifuged at 800 ×*g* for 10 min at 20° C. Collected organs and plasma supernatants were stored at -80° C until use. Plasma specimens were thawed and 100 μL plasma aliquots were transferred into 1.5 mL tubes, 300 μL methanol was added, and the mixture was vortexed for 30 s. All samples were stored at -40° C for 1 h. Samples were then vortexed for 30 s and centrifuged at 13,000 ×*g* at 4° C for 15 min. The supernatant (200 μL) and 5 μL of 140 μg/mL DL-o-chlorophenylalanine (Merck, Darmstadt, Germany) were transferred to a vial as an internal standard for LC-MS analysis.

### LC-TOF-MS

Separation was performed using an Ultimate 3000 LC combined with Q Exactive MS (Thermo Fisher Scientific, Waltham, MA, USA) and screened with ESI-MS. The LC system comprised an ACQUITY UPLC HSS T3 (100 × 2.1 mm; 1.8 μm) with an Ultimate 3000 LC. The mobile phase was composed of solvent A (0.05% formic acid-water) and solvent B (acetonitrile) with a gradient elution (1-16 min, 95–5% A; 16-18 min, 5% A; 18-19 min, 5–95% A; 19-20 min, 95–95% A). The flow rate of the mobile phase was 0.3 mL/min. The column temperature was maintained at 40° C, and the sample manager temperature was set at 4° C. Mass spectrometry parameters in ESI^+^ and ESI^-^ mode are listed as follows: ESI^+^: Heater Temp 300° C; Sheath Gas Flow rate, 45 arb; Aux Gas Flow Rate, 15 arb; Sweep Gas Flow Rate, 1 arb; spray voltage, 3.0 kV; capillary temperature, 350° C; S-Lens RF level, 30%. ESI^-^: Heater Temp 300° C, Sheath Gas Flow rate, 45 arb; Aux Gas Flow Rate, 15 arb; Sweep Gas Flow Rate, 1 arb; spray voltage, 3.2 kV; capillary temperature, 350° C; S-Lens RF level, 60%. Each sample was analyzed in triplicate.

### Statistical analysis

In the metabolomic analysis, the raw data were acquired and aligned using the Compound Discover 3.0 (Thermo Fisher Scientific) based on the m/z value and retention time of the ion signals. Ions from both ESI^-^ and ESI^+^ were merged and imported into the SIMCA-P (version 14.1, Umetrics, Umea, Sweden) for multivariate analysis. PCA was first used as an unsupervised method for data visualization and outlier identification. Supervised regression modeling was then performed on the data set using PLS-DA or OPLS-DA to identify potential biomarkers. The significantly different metabolites (ions) between the two groups were filtered out based on VIP values (VIP > 1.5). Univariate analysis, including FC analysis and *t*-tests, were performed on a volcano plot. The biomarkers were filtered and confirmed by combining the results of the VIP values (VIP > 1.5), FC values (FC > 2.0), and *t*-test (*P* < 0.05).

The data are presented as means ± S.D. values for gene expression analysis. The significance of differences in the mean was determined using a *t*-test. The threshold of statistical significance was set at *P* < 0.05.

### Compound identification

The chemical structures of important metabolites were identified using online databases, such as the Human Metabolome Database (http://hmdb.ca), Metlin (http://metlin.scripps.edu), and the Mass Bank (http://www.massbank.jp), using the data of accurate masses and MS/MS fragments. When necessary, further confirmation was acquired through comparisons with authentic standards, including retention times and MS/MS fragmentation patterns.

### Cluster analysis

Mean values of metabolite contents from biological replicates of the MTKO and WT groups were used to calculate the metabolite ratio. After log transformation of the data, the median centered ratios were normalized. Hierarchical clustering analysis (HCA) was performed using the complete linkage algorithm of the program Cluster 3.0 (Stanford University). Metabolite ratios from two independent experiments for every significant metabolite were used for HCA.

### Correlation-based network analysis of metabolites

To investigate the latent relationships of the metabolites, we constructed a correlation network diagram based on KEGG database and MBRole. All significant metabolites were imported to obtain categorical annotations, including pathways, enzyme interactions, and other biological annotations.

### RNA isolation and mRNA expression analysis

Frozen organ lysates were prepared using RNAiso Plus reagent (Takara Bio, Ohtsu, Japan). After the organs were lysed, chloroform (1/5 of the volume of RNAiso Plus reagent) was added to the samples. The samples were centrifuged (11,000 × *g*) at 4° C for 15 min, and the supernatants were collected, to which one volume of RNA-free water and 0.7 volume of ethanol were added. The lysates were then added to the Nucleospin RNA Kit (Takara Bio) columns, and total RNA was purified according to the manufacturer’s instructions. For reverse transcription, 2 μg of RNA from each sample was reverse-transcribed using a High-Capacity cDNA RT Kit (Life Technologies, Carlsbad, CA, USA) according to the manufacturer’s instructions. The cDNA samples were then subjected to real-time PCR using TB Green Premix Ex Taq II (Takara Bio) in a StepOnePlus Real-Time PCR system (Thermo Fisher Scientific). The selected primer sequences are listed in Supplementary Table 1. The PCR program was performed as follows: holding stage at 95° C for 20 s, followed by 40 cycles at 95° C for 3 s and 60° C for 30 s. Each sample was analyzed in triplicate. To verify specificity, melting curve analysis and agarose gel electrophoresis were performed on the real-time RT-PCR products. The standard curve method was used to calculate the PCR product level by plotting the quantification cycle values of a serial dilution of all cDNA sample mixtures on the y-axis against the logarithm of the standard sample concentrations on the x-axis. The relative amount of β-actin (*Actb*) mRNA as an internal control housekeeping gene was determined to compensate for variations in RT-PCR efficiency.

## Supplementary Material

Supplementary Figures

Supplementary Table 1
