# Supplementary material for: Metabolomic profiling of plasma from middle-aged and advanced-age male mice reveals the metabolic abnormalities of carnitine biosynthesis in metallothionein gene knockout mice
Source: Aging (Albany NY). 2021 Dec 1;13(23):24963–88. doi: 10.18632/aging.203731 (PMC8714139; doi:10.18632/aging.203731)
Supplement: Supplementary Figures [file aging-13-203731-s001.pdf]

SUPPLEMENTARY FIGURES

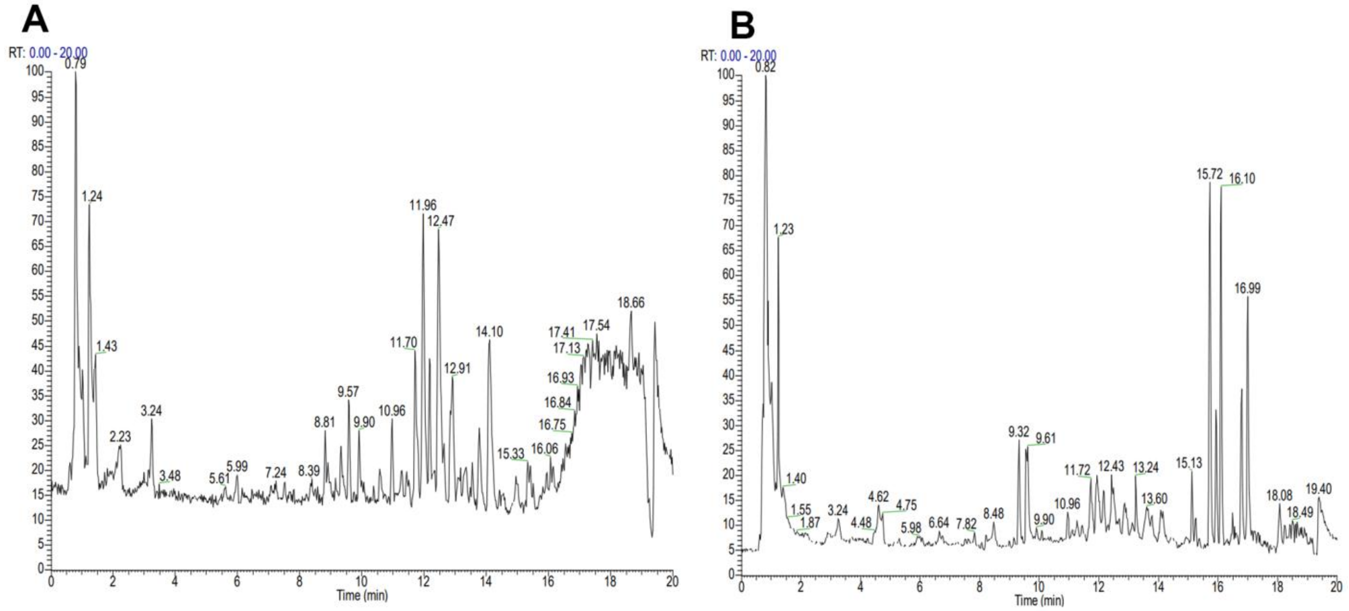

Supplementary Figure 1. Base peak intensity (BPI) chromatograms obtained from mouse plasma in liquid chromatography mass spectrometry (LC-MS). (**A**) Positive (ESI^+) scan mode, (**B**) negative (ESI^-) scan mode.

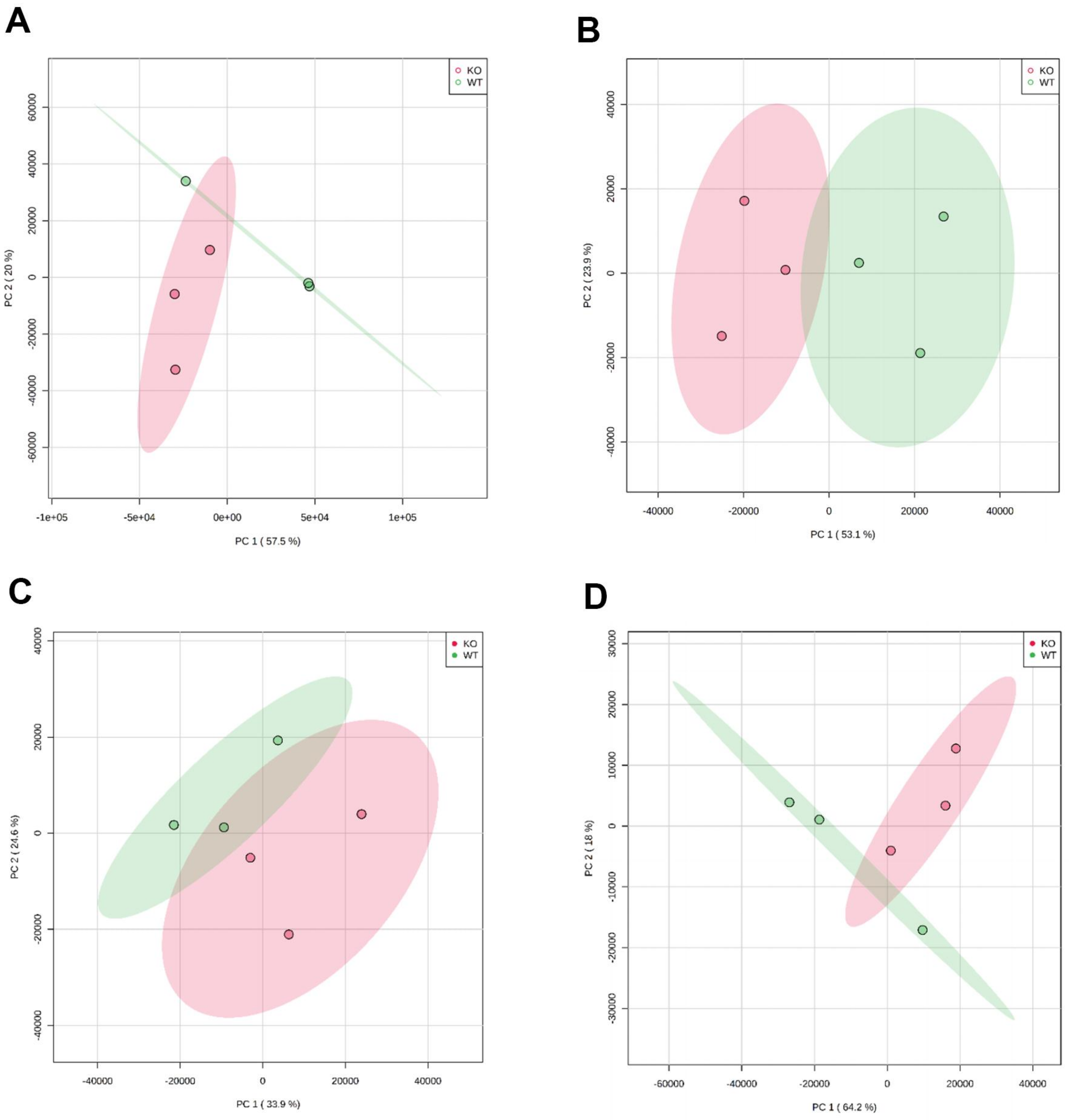

Supplementary Figure 2. Score scatter plot of PCA model. (**A**) ESI^+ scan in 50-week-old mice; (**B**) ESI^- scan in 50-week-old mice; (**C**) ESI^+ scan in 100-week-old mice; (**D**) ESI^- scan in 100-week-old mice. Magenta circles indicate MTKO mice and green circles indicate WT mice.

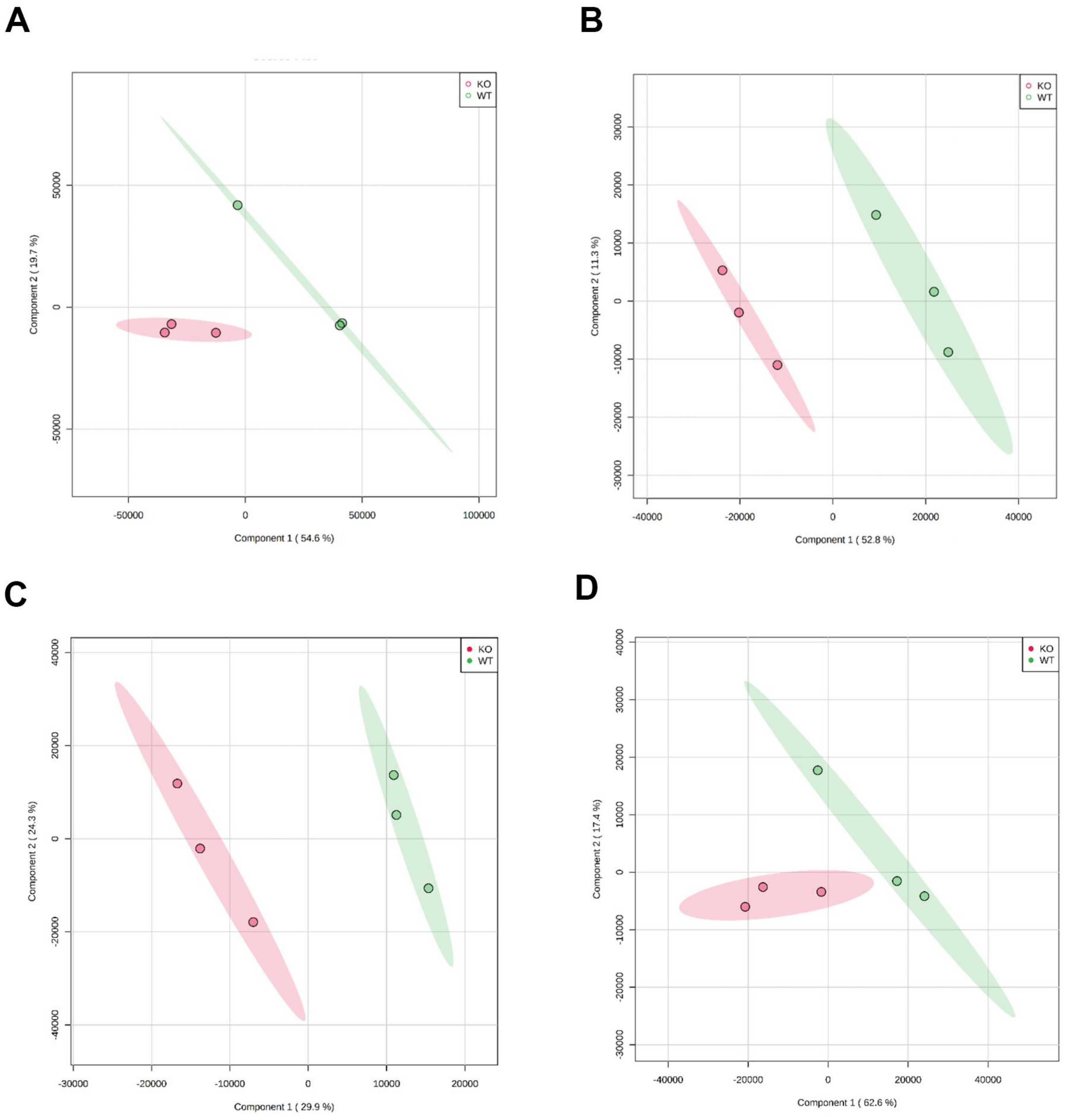

Supplementary Figure 3. Score scatter plot of PLS-DA model. (**A**) ESI$^+$ scan in 50-week-old mice; (**B**) ESI$^-$ scan in 50-week-old mice; (**C**) ESI$^+$ scan in 100-week-old mice; (**D**) ESI- scan in 100-week-old mice. Magenta circles indicate MTKO mice and green circles indicate WT mice.

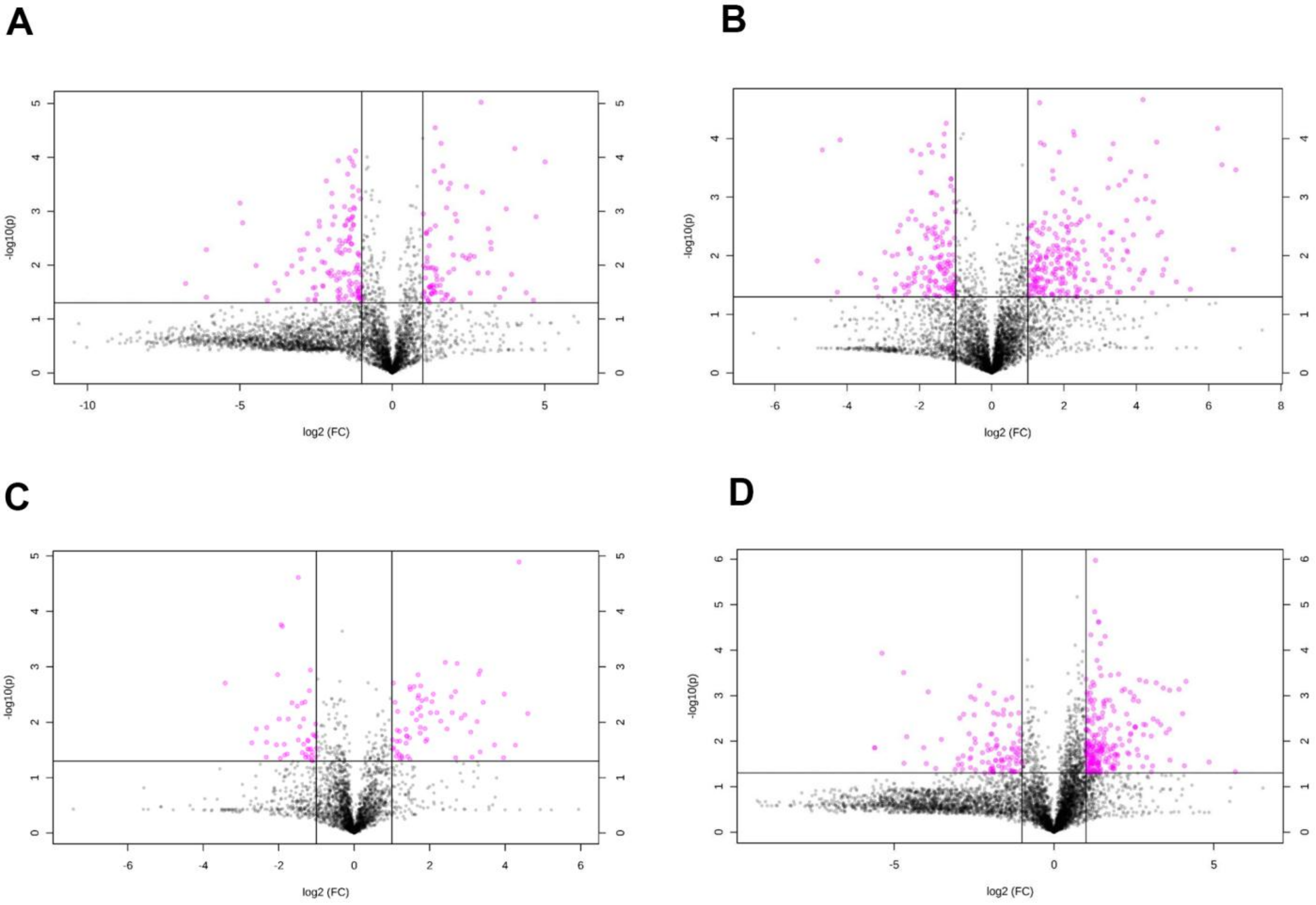

Supplementary Figure 4. Volcano plot of data. The ranges, Y > 1.30 and X > 1, indicate a significant increase; the ranges, Y > 1.30 and X < -1, indicate a significant decrease (magenta circles). (**A**) ESI$^+$ scan in 50-week-old mice; (**B**) ESI$^-$ scan in 50-week-old mice; (**C**) ESI$^+$ scan in 100-week-old mice; (**D**) ESI$^-$ scan in 100-week-old mice.

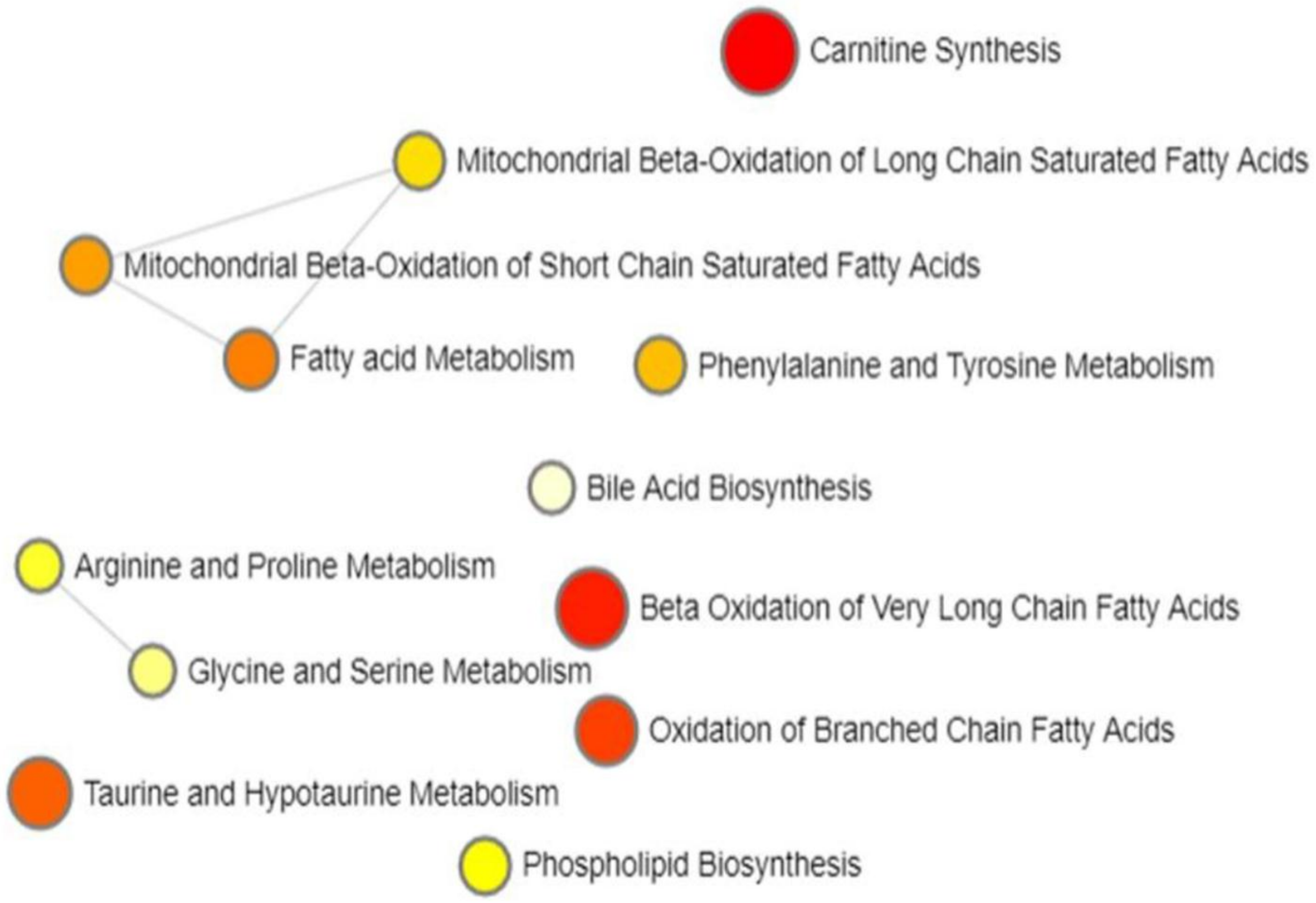

Supplementary Figure 5. A correlation network diagram obtained from pathway enrichment analysis of perturbed metabolites in 100-week old MTKO mice in ESI$^+$ mode. Each node denotes one different function, while more significant functions are indicated by a darker color.
