# Supplementary material for: Metabolomic profiling of plasma from middle-aged and advanced-age male mice reveals the metabolic abnormalities of carnitine biosynthesis in metallothionein gene knockout mice
Source: Aging (Albany NY). 2021 Dec 1;13(23):24963–88. doi: 10.18632/aging.203731 (PMC8714139; doi:10.18632/aging.203731)
Supplement: Supplementary Table 1 [file aging-13-203731-s002.pdf]

SUPPLEMENTARY TABLE

Supplementary Table 1. Primer information.

Gene	Primer sequence		Size (bp)
	Forward	Reverse	
Tmlhe	5'- CTGTGCCTTATGATGTTGTCCA -3'	5'- TGCCATGAAGTACTCGCCAG -3'	143
Tha1	5'- CCCTACCATCCAGTCTGTGAG -3'	5'- TCAACCGAGCTCCATCCA -3'	147
Shmt1	5'- ACACTGCAGATTCAGAGCCACA -3'	5'- TTGGCAAACACAGGCTGTTCCT -3'	185
Shmt2	5'- ACGCGTGTTGGAACTTGTCT -3'	5'- TCCAAGCCAATGTTGACTCCCT -3'	168
Aldh9a1	5'- CGTTGAGAATGCAAAGGCTG -3'	5'- ACTTCCCGTTGTTGATGGTC -3'	142
Bbox1	5'- AGAACCCTCAGGCTTTCCA -3'	5'- TGTTGAAGTTGACGCGAACC -3'	146
Slc22a5	5'- GCTGGGAGTACGACAAGGA -3'	5'- AAACAAGGAGGTGGTGAGTGG-3'	100
Actb	5'- GAGGCCCAGAGCAAGAGAG -3'	5'- GGCTGGGGTGTTGAAGGT -3'	225
